# Peroxiredoxin 6 Fails to Limit Phospholipid Peroxidation in Lung from *Cftr*-Knockout Mice Subjected to Oxidative Challenge

**DOI:** 10.1371/journal.pone.0006075

**Published:** 2009-06-29

**Authors:** Stéphanie Trudel, Mairead Kelly, Janine Fritsch, Thao Nguyen-Khoa, Patrice Thérond, Martine Couturier, Michal Dadlez, Janusz Debski, Lhousseine Touqui, Benoit Vallée, Mario Ollero, Aleksander Edelman, Franck Brouillard

**Affiliations:** 1 INSERM, U845, Université Paris Descartes, Faculté de Médecine, Paris, France; 2 Hôpital Necker AP-HP, Laboratoire de Biochimie A, Paris, France; 3 EA 3617, Université Paris Descartes, Faculté de Pharmacie, Paris, France; 4 Institute of Biochemistry and Biophysics, Polish Academy of Sciences, Warszawa, Poland; 5 Institute of Genetics and Biotechnology, Biology Department, Warsaw University, Warszawa, Poland; 6 Institut Pasteur, Unité de Défense Innée et Inflammation, Paris, France; 7 Plateau Protéomes IFR94, Paris, France; LMU University of Munich, Germany

## Abstract

Oxidative stress plays a prominent role in the pathophysiology of cystic fibrosis (CF). Despite the presence of oxidative stress markers and a decreased antioxidant capacity in CF airway lining fluid, few studies have focused on the oxidant/antioxidant balance in CF cells. The aim of the current study was to investigate the cellular levels of reactive oxygen species (ROS), oxidative damage and enzymatic antioxidant defenses in the lung of *Cftr*-knockout mice in basal conditions and as a response to oxidative insult.

The results show that endogenous ROS and lipid peroxidation levels are higher in *Cftr* ^−/−^ lung when compared to wild-type (*Cftr* ^+/+^) in basal conditions, despite a strong enzymatic antioxidant response involving superoxide dismutases, glutathione peroxidases and peroxiredoxin 6 (Prdx6). The latter has the unique capacity to directly reduce membrane phospholipid hydroperoxides (PL-OOH). A dramatic increase in PL-OOH levels in *Cftr* ^−/−^ lung consecutive to *in vivo* oxidative challenge by paraquat (PQ) unmasks a susceptibility to phospholipid peroxidation. PQ strongly decreases Prdx6 expression in *Cftr* ^−/−^ mice compared to *Cftr* ^+/+^. Similar results were obtained after *P. aeruginosa* LPS challenge. Two-dimensional gel analysis of Prdx6 revealed one main molecular form in basal conditions and a PQ-induced form only detected in *Cftr* ^+/+^ lung. Mass spectrometry experiments suggested that, as opposed to the main basal form, the one induced by PQ is devoid of overoxidized catalytic Cys47 and could correspond to a fully active form that is not induced in *Cftr* ^−/−^ lung. These results highlight a constitutive redox imbalance and a vulnerability to oxidative insult in *Cftr* ^−/−^ lung and present Prdx6 as a key component in CF antioxidant failure. This impaired PL-OOH detoxification mechanism may enhance oxidative damage and stress-related signaling, contributing to an exaggerated inflammatory response in CF lung.

## Introduction

Cystic fibrosis (CF), the most prevalent lethal autosomal recessive disorder in the Caucasian population, is caused by mutations in the cystic fibrosis transmembrane conductance regulator gene, *Cftr*. The latter encodes a membrane protein, CFTR, expressed in the apical membrane of exocrine epithelial cells that functions as a cyclic AMP-activated anion channel. Although CF is a multisystem disease with a typical phenotype characterized by pancreatic insufficiency, intestinal obstruction and male infertility, the lung disease remains as its most life-threatening manifestation. CF lung disease is regarded as a combination of abnormalities in electrolyte transport, epithelial lining fluid (ELF) composition, pathogen clearance and inflammatory response that set in motion a self-perpetuating cycle of airway obstruction, chronic bacterial infection and inflammation.

CF airways inflammation appears excessive and prolonged in regard to bacterial load and associated with large numbers of neutrophils. This chronic inflammation leads to fibrosis and the progressive deterioration of pulmonary function in CF. Several studies indicate that oxidant/antioxidant imbalance and oxidative stress are important players in this scenario. Elevated levels of lipid and protein oxidation products found in BALF, exhaled breath condensate and sputum of CF patients [1] [2] [3] [4], provide evidence of oxidative stress in the ELF compartment. These oxidative damages are due to an increased oxidant burden, resulting from the release of oxidants by neutrophils and *Pseudomonas aeruginosa* that chronically infects CF airways [5]. Concentrations of glutathione in the ELF are markedly reduced in CF patients as well as *Cftr*-knockout mice [6] [7], contributing to the oxidant/antioxidant imbalance. It has been postulated that CFTR, by participating in the apical export of reduced glutathione (GSH) [8] [9] [10], is directly involved in the regulation of GSH homeostasis of ELF. Thereby, CFTR could be considered as an important actor of ELF antioxidant homeostasis and thus an intrinsic cause of oxidative imbalance in CF airways from human patients as well as *Cftr* knockout mice. The latter appears as a suitable model to investigate the constitutive redox imbalance in CF.

To minimize the damage on their constituents, cells rely on the scavenging capacity of reactive oxygen species (ROS) by enzymatic (*i.e.* superoxide dismutases, catalase, GSH-peroxidases) and non-enzymatic antioxidant systems. The levels of antioxidant enzyme expression and function are supposed to reflect the capacity of tissues to protect from oxidant-induced injury and are widely considered as important parameters to evaluate oxidative stress. Among antioxidant enzymes, peroxiredoxin 6 (Prdx6) has been recognized as a major player in the defence against lung oxidative damage [11] [12] [13]. Prdx6 has a single redox-active cysteine and uses glutathione as the electron donor to catalyze the reduction of H_2_O_2_, fatty acid hydroperoxides and more interestingly phospholipid hydroperoxides (PL-OOH) [14]. PL-OOH can propagate an autocatalytic chain of lipid peroxidation and generate toxic secondary products such as malonyldialdehyde (MDA) and 4-hydroxyalkenals that are prone to form oxidative adducts and activate stress signalling pathways. The unique ability of Prdx6 to reduce membrane PL-OOH probably explains its crucial role in lung antioxidant defence [15].

Although several lines of evidence indicate that pro-oxidative conditions of ELF exacerbate deterioration of airways in CF, there is comparatively little information about the intracellular redox status in CF airway cells. The aim of the current study was to evaluate (i) the enzymatic antioxidant activities, (ii) lipid peroxidation levels, and (iii) the *in vivo* capacity of CF lung to respond to oxidative insult in a *Cftr*-knockout mouse model. The results presented here show a constitutive redox imbalance and a vulnerability to oxidative stress involving Prdx6 in *Cftr* ^−/−^ lung.

## Methods

### Reagents

Media, serum and antibiotics for cell culture were purchased from Invitrogen (GIBCO®). Chemicals for protein concentration assessment, gel electrophoresis and transfer were purchased from Biorad (Paris, France), primary antibodies from Abcam (Paris, France) and Odyssey reagents from ScienceTec (Paris, France).

### Animals

All animal protocols were approved by Necker Faculty of Medicine Animal Care and Use Committee (Université Paris Descartes); authorization no. 7514, Ministère de l'Agriculture et de la Pêche. Animals used in this study were Cftr-knockout (Cftr −/−) mice homozygous for the S489X mutation (B6;129-Cftrtm1Unc) [16] backcrossed into C57BL/6 (three generations) and their wild-type (Cftr+/+) littermates (CDTA, Orléans, France). Pups were weaned at 21 days and fed solid mouse chow (Teklad 2018S, Harland). Cftr −/− mice typically die shortly after weaning from intestinal obstruction. A commercial osmotic laxative containing PolyEthylenGlycol-3350 and electrolytes (Movicol®, Norgine Pharma, Paris, France) was added to the drinking water (1pack/250 ml) to increase the survival of Cftr −/− mice [17]. All mice were allowed food and water ad libitum until the time of sacrifice by cervical dislocation.

### Paraquat (PQ)-induced oxidative stress in mice

Mice (4 week-old) were injected twice (intraperitoneally) at 24 hours intervals with paraquat (methylviologen dichloride hydrate, Sigma-Aldrich, St-Quentin, France) with doses of 30 mg/kg of body weight in normal saline (sodium chloride 0.9%, MacoPharma, Mouvaux, France). Mice were sacrificed 48 hours after the first injection.

### Lipopolysaccharide (LPS) instillation

Mice (5–7 week-old) were slightly anesthetized with xylazine 2% (8 mg/kg) (Rompun®, Bayer-France) and ketamine 1000 (40 mg/kg) (Imalgène 1000, Merial, Lyon, France) and received intratracheal instillation of *P. aeruginosa* LPS (330 µg/kg) (serotype 10; Sigma-Aldrich, St-Quentin, France). Mice were sacrificed 24 hours later.

### Harvesting and preparation of mice tissues

Tracheas were incised and cannulated to allow bronchoalveolar lavage. Lung lobes were lavaged three times by instillation and aspiration of 500 µl of sterile PBS pH 7.4. Right and left lungs were rinsed with cold PBS and rapidly frozen in liquid nitrogen and stored at −80°C for subsequent measurement of thiobarbituric acid-reactive substances. For phospholipid hydroperoxide quantification, lungs were homogenized in 500 µl of PBS before lipid extraction. For immunohistochemistry experiments, lungs were filled with PBS containing 50% Shandon Cryomatrix™ (Thermo Scientific, Cergy-Pontoise, France) and immediately frozen in liquid nitrogen.

### Isolation of tracheal cells and fluorescent measurement of intracellular oxidant

Tracheas were dissected away from lungs and collected in ice-cold DMEM:F-12 medium with 100 U/ml penicillin and 100 µg/ml streptomycin before proceeding to epithelial cell isolation. This was performed as reported by You *et al* [18] with slight modifications. Tracheas were opened longitudinally and incubated in 1 ml of DMEM:F-12 with 100 U/ml penicillin and 100 µg/ml streptomycin containing 1.5 mg/ml of pronase (Roche applied science, Meylan, France) for 18 h at 4°C. After adding foetal calf serum (FCS) up to a final concentration of 10%, tracheas were inverted 12 times to release cells and transferred to another tube. The process was repeated twice, the three tubes were pooled and centrifuged at 400 *g* for 10 min at 4°C. Cells were resuspended in 200 µl DMEM:F-12 medium with 10% FCS per trachea. At this step, cell suspensions were pooled to have a sufficient amount of ciliated cells for quantification. Cells were seeded in tissue culture plates for 3–4 h in 5% CO_2_ at 37°C to remove fibroblasts. Non-adherent cells were collected by centrifugation and resuspended in Ringer solution (10 mM Hepes, 5 mM KCl, 135 mM NaCl, 1 mM MgCl_2_, 1 mM CaCl_2_ and 10 mM glucose). Cells were then incubated for 30 min at 37°C with 1 µM of the ROS sensitive fluorescent probe, 5-(and-6)-chloromethyl-2′,7′-dichlorodihydrofluorescein diacetate (CM-H_2_DCFDA, Molecular Probes, Cergy-Pontoise, France). The incubation was performed in Lab-Tek® chamber mounted on glass slide (Nunc, Thermo Scientific, Cergy-Pontoise, France), previously coated with BD Cell-Tak™ (BD Biosciences, Le-Pont-de-Claix, France) to adhere the cells. After incubation, cells were washed twice with Ringer solution. Images were obtained by laser confocal scanning microscopy (Zeiss LSM 510) with ×63 oil objectives. Only ciliated tracheal cells were used for quantification of relative fluorescence with ImageJ software (rsb.info.nih.gov/ij).

### Thiobarbituric acid-reactive substances (TBARS) assay

Malondialdehyde, one of the several low molecular weight end products formed via the decomposition of certain primary and secondary lipid peroxidation products, can be measured by reaction with thiobarbituric acid [19]. Lipid peroxidation was determined by measuring the formation of TBARS using a commercial assay kit (Cayman Chemical, Ann Arbor, MI, USA) in accordance with the manufacturer's protocol. TBARS concentration was calculated from a MDA standard curve and normalized for protein content.

### Determination of total superoxide dismutase (SOD) activity

Lung tissue was homogenized (Dounce homogenizer) in 500 µl of ice-cold buffer (20 mM Hepes pH 7.2, 1 mM EGTA, 210 mM mannitol, 70 mM sucrose), centrifuged (1500 *g* for 5 min at 4°C), and the supernatant was retained for analysis. Total SOD activity was determined using a commercially available kit (Cayman Chemical, Ann Arbor, MI, USA) [20] [21]. SOD specific activity was expressed as units per gram of lung protein.

### Determination of total glutathione peroxidase (GPx) activity

Lung tissues were homogenized (Dounce homogenizer) in 750 µl of cold buffer (50 mM Tris-HCl pH 7.5, 150 mM NaCl, 1 mM EDTA, 0.5% Triton® X-100), centrifuged (10 000 *g* for 10 min at 4°C) and the supernatant was retained for analysis. Total GPx activity in the homogenate was measured according to the protocol supplied with the kit (Cayman Chemical, Ann Arbor, MI, USA). GPx specific activity is expressed as μmole of NADPH consumed per minute per milligram of lung protein.

### Determination of Catalase (CAT) activity

Lung tissues were homogenized (Dounce homogenizer) in 500 µl of ice-cold buffer (50 mM potassium phosphate pH 7.0, 1 mM EDTA), centrifuged (10 000 *g* for 15 min at 4°C) and the supernatant was retained for analysis. CAT activity was determined using a commercially available kit (Cayman Chemical, Ann Arbor, MI, USA) [22], [23] CAT specific activity was expressed as nmole of formaldehyde formed per minute per mg of lung protein.

### Immunoblot analysis

Ten micrograms of mice lung homogenates were diluted with Laemmli sample buffer and heated at 95°C for 3 min. Proteins were resolved by 12% SDS-polyacrylamide gel electrophoresis and electrotransferred onto a PVDF membrane. After transfer, analysis was performed following manufacturer's recommendations for the Odyssey® infrared imaging system (LI-COR Biosciences, NE, USA). Dilution of rabbit polyclonal antibodies to Prdx6 (Abcam, Paris, France) was 1∶2,000 and 1∶10,000 for the fluorescent IRDye® TM700 secondary antibody (ScienceTec, Paris, France). Images were acquired with the Odyssey infrared imaging system. Quantification of protein bands was performed with the Odyssey software. Membranes were also probed with mouse monoclonal antibody to α-tubulin from AbCam (1∶10,000) and IRDye® TM800 secondary antibody from ScienceTec (1∶10,000) to correct for protein loading.

### Immunohistochemical staining

Six-micrometer cryosections of mouse tissues were fixed with cold acetone for 10 min at 4°C. All steps were carried out in a humid chamber. Sections were rehydrated in PBS and permeabilized with 0.25% Triton® X-100 in PBS. Nonspecific binding sites were blocked with 3% bovine serum albumin in 0.1% Triton® X-100 in PBS (PBS-T) before incubation with a dilution of Prdx6 primary antibody (1∶1,200) in 1% bovine serum albumin in PBS-T. After washing and blocking with 5% goat serum in PBS-T, sections were incubated with Alexa Fluor® 594 goat anti-rabbit secondary antibody (Molecular Probes, Cergy-Pontoise, France) diluted 1∶1,000 in PBS-T and subsequently mounted in Vectashield mounting medium (Abcys, Paris, France). Tissue sections were examined under a confocal laser scanning microscope (Zeiss LSM 510). Images were collected with ×40 or ×63 oil objectives.

### Quantitative Real-Time PCR

Total RNAs were isolated using TRIzol reagent following manufacturer's recommendations (Invitrogen, Cergy-Pontoise, France), dissolved in RNAse-free water and measured at 260 nm. Gene expression was studied by quantitative Real-Time PCR using the cycle threshold (CT)-based method [24]. Five μg of total RNA was used to synthesize cDNA (First-strand cDNA Synthesis Kit, GE Healthcare, Orsay, France) with random hexadeoxynucleotides and reverse transcriptase for 1 h at 37°C. The LightCycler FastStart DNA Master PLUS SYBR Green I kit was used with a LightCycler (Roche applied science, Meylan, France) for quantitative PCR. The relative expression of each target gene was normalized to a reference gene, β-actin. Gene specific primers were chosen using Primerbank (pga.mgh.Harvard.edu/primerbank). The primers for Prdx6 were forward: CGCCAGAGTTTGCCAAGAG and reverse: TCCGTGGGTGTTTCACCATTG. The primers for β-actin were forward: AGTGTGACGTTGACATCCGTA and reverse: GCCAGAGCAGTAATCTCCTTCT.

### Phosphatidylcholine hydroperoxide (PC-OOH) quantification

Lung wet weight was 0.14 g±0.01 g for Cftr^−/−^ and 0.23 g±0.01 g for Cftr^+/+^ mice. Lung tissue lipids were extracted by adding 3 ml of chloroform∶methanol 12∶1 (vol/vol) to the whole lung homogenate, vortexing for 10 sec and centrifuging at 800 *g* for 5 min at 4°C. The lower organic phase was collected and evaporated to dryness under a nitrogen stream. Separation and detection of phosphatidylcholines (PC) and their corresponding hydroperoxides (PC-OOH) were performed as previously described [25]. Briefly, the entire extract of dried lipids was dissolved in methanol and then injected for HPLC separation, using an RP-18 (Kromasil 250×4.6 mm, 5 µm particles) analytical column maintained at 40°C. The mobile phase was composed of 6% 10 mM ammonium acetate pH 5.0 and 94% methanol. The molecular species of PC were detected at 205 nm and PC-OOH were detected by a Jasco CL-925 chemiluminescence detector (Japan Spectroscopic Co, Tokyo, Japan) in a post-column reaction: eluting hydroperoxides reacted with the chemiluminescent reagent prepared by dissolving microperoxidase (10 mg/l) and isoluminol (55 mg/l) in a mixture of borate buffer (0.1 M, pH 9.2, 300∶700 vol/vol). Peaks corresponding to molecular species of PC and PC-OOH were identified from the same HPLC injection by their retention time relative to standards: PC standards were purchased from Avanti Polar Lipids (Alabaster, AL, USA) and their corresponding PC-OOH were prepared by lipoxygenase treatment [26]. Molecular species of PC-OOH (1-palmitoyl-2-linoleoyl-phosphatidylcholine (PLPC) [16∶0-18∶2-OOH], 1-palmitoyl-2-arachidonoyl-phosphatidylcholine (PAPC) [16∶0-20∶4-OOH] and 1-palmitoyl-2-docosahexaenoyl-phosphatidylcholine (PDPC) [16∶0-22∶6-OOH] were quantified by their peak height normalized to the peak height of the corresponding non-peroxidized PC. As PAPC-OOH and PDPC-OOH had the same retention time they were quantified together.

### Determination of protein carbonyls

The content of carbonyl group in lung protein was assessed by the ELISA method using a commercially available kit (Immundiagnostik AG, Bensheim, Germany) in accordance with the manufacturer's protocol. Lung tissue was homogenized (Dounce homogenizer) in 500 µl of ice-cold PBS, centrifuged (10 000 *g* for 10 min at 4°C), and the supernatant was retained for analysis. The carbonyl protein content was calculated from a standard curve prepared with oxidized bovine serum albumin and normalized for protein content.

### Two-dimensional (2D) gel electrophoresis

The 2D gel techniques were adapted from Bensalem *et al* [27]. Aliquots of mouse lung homogenates were resolubilized in buffer containing 7 M urea, 2 M thiourea, 4% CHAPS, 0.5% dithiothreitol and 1% ampholyte pH 4–7. Samples were applied by in-gel rehydration (50 V, 10 h) and isoelectrofocusing (IEF) was performed on immobilized pH gradient strips (pH 4–7, GE Healthcare, Orsay, France). Seven cm strips (25 µg protein load, 125 µl rehydration volume) were used with gels for immunoblotting and 18 cm strips (350 µg, 450 µl) were used with gels for mass spectrometry. IEF was performed for a total of ∼16 kVh (immunoblotting) or ∼60 kVh (mass spectrometry). Prior to SDS-PAGE on a 12% polyacrylamide gel, strips were incubated at room temperature in equilibration buffer (50 mM Tris-HCl pH 8.8, 6 M urea, 2% SDS) with 2% DTT for 10 min and for another 10 min with 2.5% iodoacetamide. Immunoblotting with Prdx6 antibodies was performed as described above. Gels for mass spectrometry were fixed, washed and proteins were visualized by the silver staining method as previously described [28].

### Mass spectrometry and *Database Searching*

The peptide mixture obtained by the standard in-gel tryptic digestion procedure (including reduction and alkylation) was applied to an RP-18 precolumn (Waters NanoAcquity 20 mm×180 µm) using water containing 0.1% trifluoroacetic acid as a mobile phase and then transferred to a UPLC RP-18 column (Waters NanoAcquity 250 mm×75 µm) using an acetonitrile gradient (0–50% in 30 min) in the presence of 0.1% formic acid at a flow rate of 250 nL/min. The column outlet was directly coupled to the ion source of the Ion Cyclotron Resonance spectrometer (LTQ-FTICR, Thermo Electron), working in the regime of data-dependent MS to MS/MS switch. The resulting mass spectra were used to search the nonredundant protein database of the National Center of Biotechnology Information (NCBInr version 20080624) using the MASCOT (matrixscience.com) search engine (8-processor on-site license). Standard search parameters were set as follows: taxonomy restriction – *Mus musculus*, enzyme – semi-trypsin, variable modifications – carbamidomethylation (C), oxidation (M), protein mass – unrestricted, peptide mass tolerance −±40 ppm, fragment mass tolerance −±0.8 Da, max missed cleavages −1. A second search was performed using the error tolerant function of Mascot. This mode allows iterating selected proteins through a list of chemical and post-translational modifications present in the UniMod database (www.unimod.org). Potential modifications were validated by manual inspection of the MS/MS data. Hits were accepted if all strong peaks in the MS/MS spectrum were assigned either to y- or b-series ions and the spectrum contained y- or b-series signals corresponding to at least a stretch of three or more consecutive amino acids.

### Statistical analysis

Unpaired Student's *t* test was used for comparison of means and *P* values ≤0.05 were considered statistically significant. Data were expressed as mean±S.E.

## Results

### Abnormal oxidant/antioxidant status in airway cells from *Cftr* ^−/−^ mice

**Figure 1:**
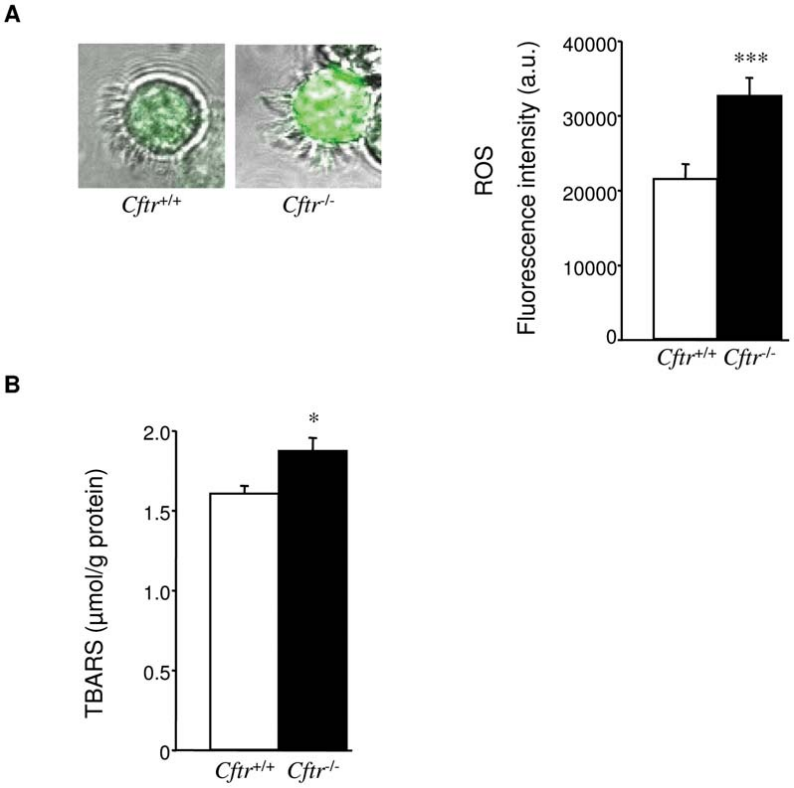
Intracellular ROS and lipid peroxidation levels. *A) Endogenous ROS levels in ciliated tracheal cells*. Epithelial cells freshly isolated from trachea were incubated with CM-H2DCF-DA and visualized by fluorescent confocal microscopy. Under identical imaging conditions, DCF fluorescence was markedly increased in ciliated epithelial cells from *Cftr* ^−/−^ mice (left) as confirmed by quantification of relative fluorescence (right). Values represent fluorescent intensity (arbitrary units, a.u.) and data correspond to the mean±S.E. of n = 90 for *Cftr* ^+/+^ and n = 71 for *Cftr* ^−/−^ cells, obtained from five animals of each genotype. *B) Lipid peroxidation in lung tissue*. Levels of TBARS were significantly elevated in lung tissue from *Cftr* ^−/−^ mice. Values are presented as malondialdehyde equivalences (micromole per gram of lung protein), mean±S.E., n = 9 from 2 different experiments, in duplicate). * P≤0.05, *** P≤0.001

The intracellular oxidant status of freshly isolated tracheal cells from *Cftr* ^−/−^ mice and their *Cftr* ^+/+^ littermates was analyzed using the fluorescent indicator CM-H_2_DCFDA. As shown in Fig. 1A, the level of DCF fluorescence was 1.5 times greater (*P* = 0.0005) in *Cftr* ^−/−^ ciliated cells when compared to *Cftr* ^+/+^, revealing a higher steady state level of intracellular ROS in *Cftr* ^−/−^ airway epithelial cells.

To investigate whether this elevated level of intracellular ROS in *Cftr* ^−/−^ cells was associated with an increased level of oxidation-damaged macromolecules, we evaluated lipid peroxidation in lung homogenates using the TBARS assay (Fig. 1B). TBARS levels were slightly but significantly higher (*P* = 0.014) in *Cftr* ^−/−^ mice when compared to *Cftr* ^+/+^.

**Figure 2:**
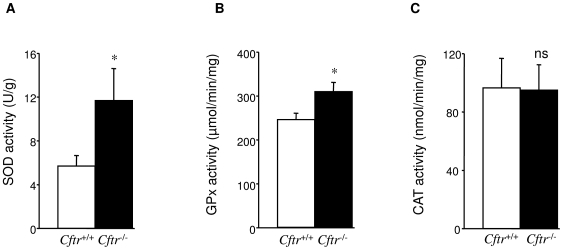
Antioxidant enzymatic activities. Comparison between *Cftr* ^+/+^ and *Cftr* ^−/−^ mice of SOD (A), GPx (B) and CAT (C) activities in lung homogenates. Enzymatic activities were measured by standard spectrophotometric methods. Lung homogenates from *Cftr* ^−/−^ mice displayed significantly higher SOD and GPx activities than those from *Cftr* ^+/+^ mice. Both *Cftr* ^+/+^ and *Cftr* ^−/−^ mice displayed a similar level of CAT activity. Values are presented as specific activity (mean±S.E., n = 5, in duplicate). * P≤0.05

To determine whether decreased antioxidant defences could account for the increased levels of ROS and peroxidized lipids in *Cftr* ^−/−^ lung, the activities of the main antioxidant enzymes in lung, e.g., SOD, GPx and CAT, were evaluated. No difference in CAT activity was measured between *Cftr* ^−/−^ and *Cftr* ^+/+^ (Fig. 2A). In contrast, total SOD and GPx activities exhibited a significant increase (*P* = 0.05 and *P* = 0.044) in *Cftr* ^−/−^ lung (Fig. 2B and C). While GPx activity presented a small increase, SOD activity was almost double in *Cftr* ^−/−^ lung extracts.

**Figure 3:**
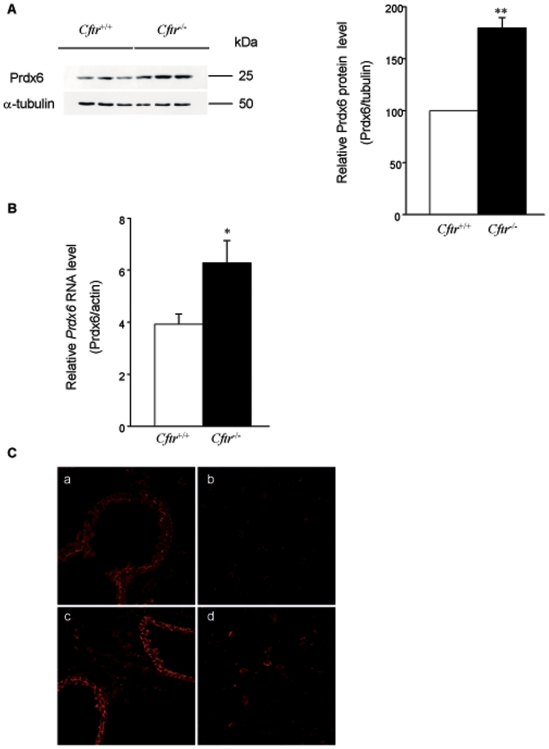
Prdx6 protein and mRNA levels. *A) Prdx6 protein expression levels*. Lung protein extracts from *Cftr* ^+/+^ and *Cftr* ^−/−^ mice were subjected to immunoblot analysis using antibodies against Prdx6 (top) and α-tubulin (bottom). On the left, a representative blot obtained with three pairs of mice is presented. It shows that Prdx6 expression levels are higher in *Cftr* ^−/−^ lung compared to *Cftr* ^+/+^, as confirmed on the right by quantitative analysis. Individual data were quantified as ratio of fluorescence intensity for Prdx6 bands to the intensity obtained for α-tubulin. Data are shown as percent of *Cftr* ^+/+^ (mean±S.E., n = 9 from 2 different experiments). *B) Prdx6 mRNA levels*. Total lung RNA from *Cftr* ^+/+^ and *Cftr* ^−/−^ mice was reversely transcribed and the amounts of cDNAs coding for Prdx6 and the reference protein β-actin were measured by quantitative Real-Time PCR. Prdx6 mRNAs are increased in *Cftr* ^−/−^ lung compared to *Cftr* ^+/+^. Indicated values represent the ratio of Prdx6 mRNAs to β-actin mRNAs (mean±S.E., n = 8, in triplicate). *C) Immunohistochemical analysis of Prdx6*. Acetone-fixed lung cryosections from *Cftr* ^+/+^ and *Cftr* ^−/−^ mice were incubated with an anti-Prdx6 antibody and visualized by confocal microscopy. Sections of bronchioles from *Cftr* ^+/+^ (a) and *Cftr* ^−/−^ (c) mice and the alveolar region from *Cftr* ^+/+^ (b) and *Cftr* ^−/−^ (d) mice are shown. *Cftr* ^−/−^ lung sections present higher Prdx6 staining intensity compared to *Cftr* ^+/+^ mice. * P≤0.05, ** P≤0.01

The presence of elevated ROS and lipid peroxidation levels despite higher total GPx and SOD activities in *Cftr* ^−/−^ raised the possibility that some important antioxidant defences could be defective in *Cftr* ^−/−^ airway cells. As Prdx6 is a major lung antioxidant and a unique phospholipid hydroperoxide detoxification system in this tissue, we further investigated its expression level in *Cftr* ^−/−^ lung. As shown in Fig. 3A, a 1.8-fold increase (*P* = 0.0021) in Prdx6 protein expression was measured in *Cftr* ^−/−^ lung by quantitative immunoblot. In contrast, no difference in Prdx6 expression was observed in skeletal muscle, a tissue where CFTR is not expressed (data not shown).

Quantitative RT-PCR analysis showed a 1.6-fold increase (*P* = 0.024) in *Prdx6* mRNA in *Cftr* ^−/−^ as compared to *Cftr* ^+/+^ lung (Fig. 3B).

Immunofluorescence analysis revealed an abundant Prdx6 staining in bronchiolar epithelial cells (Fig. 3C). A subset of alveolar cells, probably corresponding to type II pneumocytes or macrophages were also stained but to a lesser extent. The Prdx6 distribution pattern was similar in both types of mice, but staining intensity was markedly increased in *Cftr* ^−/−^ lung sections, which is consistent with immunoblotting results.

All together, these results showed that airway cells from *Cftr* ^−/−^ mice exhibit modifications of their oxidant and antioxidant status together with a slightly increased lipid peroxidation, providing evidence of moderate oxidative stress in the lung of mice lacking CFTR, before any sign of chronic inflammation.

### PC-OOH accumulation and increased protein carbonyl in PQ-challenged *Cftr* ^−/−^ lung

**Figure 4:**
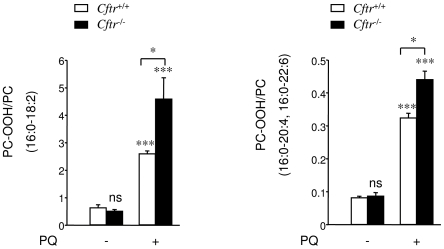
Phosphatidylcholine hydroperoxides quantification. *Cftr* ^+/+^ and *Cftr* ^−/−^ mice were injected I.P. twice with paraquat (PQ) (+) or saline (−) at 24 h intervals and sacrificed 48 h after the first injection. After lipid extraction, phosphatidylcholines (PC) and their corresponding hydroperoxides (PC-OOH) were separated by HPLC and detected by UV and chemiluminescence. After PQ challenge PLPC-OOH (16∶0–18∶2), PAPC–OOH (16∶0–20∶4) and PDPC–OOH (16∶0–22∶6) were significantly higher in *Cftr* ^−/−^ lung compared to *Cftr* ^+/+^. Data are expressed as the ratio of PC–OOH to PC peak heights (mean±S.E.) and are representative of 3 different experiments with at least n = 3 animals in each experiment. * P≤0.05, *** P≤0.001

The observed increase in Prdx6 expression in *Cftr* ^−/−^ lung prompted us to examine their ability to control phospholipid peroxidation when exposed to an oxidative challenge *in vivo*. For this purpose, mice were treated with paraquat (PQ), known to be selectively accumulated in the lung [29], where it leads to ROS generation and initiates membrane damaging processes *via* lipid peroxidation. Mice were injected I.P. twice at a 24 h interval with either PQ or saline. Lungs were analysed 48 h after the first injection for their content in phosphatidylcholine hydroperoxides (PC-OOH) by HPLC and chemiluminescence detection. The treatment did not compromise the survival of *Cftr* ^−/−^ mice (not shown). Basal levels of PC-OOH were low and not significantly different in untreated *Cftr* ^−/−^ *vs Cftr* ^+/+^ mice (Fig. 4). As expected, PQ administration induced a significant increase in PC-OOH content in both types of mice. Interestingly, for the same PQ load, the PC-OOH content was significantly higher in *Cftr* ^−/−^ lung as compared to *Cftr* ^+/+^ (*P* = 0.043 for PAPC-OOH (Fig. 4, left) and *P* = 0.021 for PAPC/PDPC-OOH (Fig. 4, right)). PQ treatment resulted in a 9- (*P* = 0.001) and 4-fold (*P* = 0.0002) increase of PLPC-OOH levels in *Cftr* ^−/−^ and *Cftr* ^+/+^ lungs, respectively. For PAPC/PDPC-OOH -fold increases were 5 (*P* = 0.0001) for *Cftr* ^−/−^ and 4 (*P* = 0.0001) for *Cftr* ^+/+^.

Protein carbonyls are formed by a variety of oxidative mechanism and are hallmarks of oxidation status of proteins. We evaluated if the paraquat challenge resulted in changes in the level of protein carbonyls. The results from the ELISA assay showed that carbonyl content was higher in *Cftr* ^−/−^ lung (234.2±6.8 pmol/mg protein) as compared to *Cftr* ^+/+^ (194.8±10.6 pmol/mg protein, n = 5, *P* = 0.014) after the treatment with PQ.

### Decreased Prdx6 expression in PQ-challenged *Cftr* ^−/−^ lung

**Figure 5:**
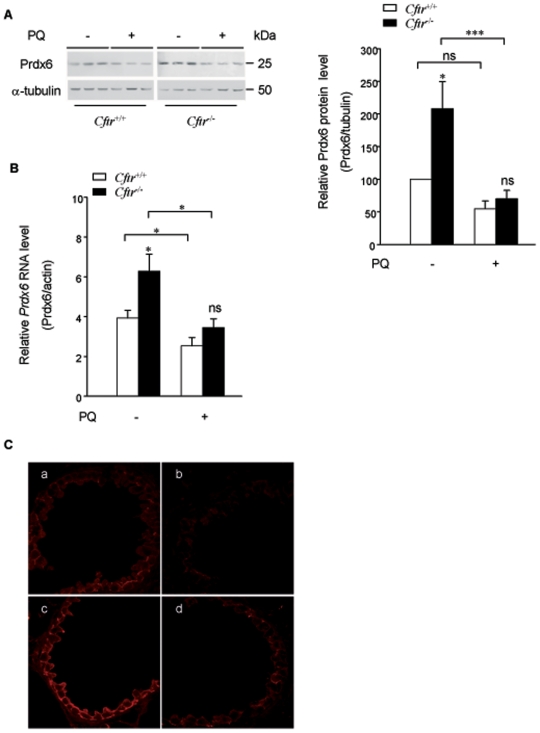
Prdx6 protein and mRNA levels after paraquat exposure. *A) Prdx6 protein expression levels after paraquat (PQ) treatment*. Lung protein extracts from *Cftr* ^+/+^ and *Cftr* ^−/−^ mice treated with either PQ (+) or saline (−) were subjected to immunoblot analysis using antibodies against Prdx6. The decrease in Prdx6 protein after PQ exposure is greater in *Cftr* ^−/−^ mice than in *Cftr* ^+/+^. On the left, a representative blot is presented. Individual data were quantified as ratio of fluorescence intensity for Prdx6 bands to the intensity obtained for α-tubulin. Data are shown as percent of *Cftr* ^+/+^ treated with saline (mean±S.E., n = 5 for saline and n = 10 for PQ). *B) Prdx6 mRNA levels after PQ treatment*. Total lung RNA from *Cftr* ^+/+^ and *Cftr* ^−/−^ mice treated with either PQ (+) or saline (−) was reversely transcribed and the amount of cDNAs coding for Prdx6 and the reference protein β-actin were measured by quantitative Real-Time PCR. The decrease in Prdx6 mRNA after PQ challenge is greater in *Cftr* ^−/−^ mice than in *Cftr* ^+/+^. Indicated values correspond to the ratio of Prdx6 to β-actin mRNA (mean±S.E., n = 8 for saline and n = 5 for PQ, in triplicate). *C) Immunohistochemical analysis of Prdx6 after PQ* treatment. Acetone-fixed cryosections from *Cftr* ^+/+^ and *Cftr* ^−/−^ mice treated with PQ or saline were incubated with anti-Prdx6 antibodies and visualized by confocal microscopy. Panels correspond to sections of bronchioles from *Cftr* ^+/+^ mice treated with either saline (a) or PQ (b) and from *Cftr* ^−/−^ mice treated with saline (c) or PQ (d). Prdx6 staining intensity was lower after PQ exposure. * P≤0.05, *** P≤0.001

Next, we evaluated Prdx6 expression after PQ treatment (Fig. 5). Immunoblot analysis revealed that Prdx6 protein expression was decreased after PQ in both *Cftr* ^−/−^ and *Cftr* ^+/+^, with a much more pronounced -fold decrease in *Cftr* ^−/−^ mice, 3-fold (*P* = 0.001) *vs* 1.8-fold (*P* = 0.094) for *Cftr* ^+/+^, revealing that both mice presented comparable Prdx6 protein levels after PQ treatment (Fig. 5A).

As shown in Fig. 5B, Prdx6 protein expression parallels its mRNA levels, as revealed by quantitative RT-PCR analysis. PQ treatment resulted in a 1.8- (*P* = 0.047) and 1.6-fold decrease (*P* = 0.05) of *Prdx*6 mRNA levels in *Cftr* ^−/−^ and *Cftr* ^+/+^ lung, respectively. Immunohistochemistry analysis showed a decreased intensity in Prdx6 staining after PQ challenge in both *Cftr* ^+/+^ and *Cftr* ^−/−^, confirming immunoblot results (Fig. 5C). Moreover, it showed that PQ does not induce any noticeable change in the cellular and subcellular localization of Prdx6.

### Decreased Prdx6 expression in lung from *Cftr* ^−/−^ mice exposed to *P. aeruginosa* LPS

**Figure 6:**
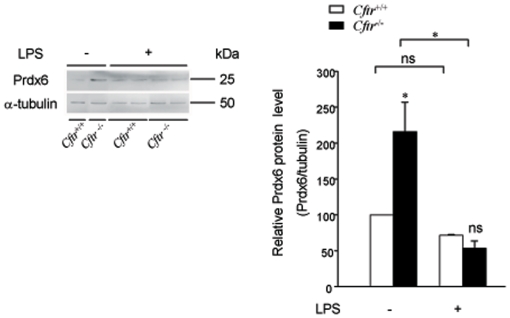
Prdx6 protein expression levels after LPS treatment. Lung protein extracts from *Cftr* ^+/+^ and *Cftr* ^−/−^ mice treated with either LPS (+) or saline (−) were subjected to immunoblot analysis using antibodies against Prdx6. The decrease in Prdx6 protein after LPS exposure is greater in *Cftr* ^−/−^ than in *Cftr* ^+/+^ lung. On the left, a representative blot is presented. Individual data were quantified as ratio of fluorescence intensity for Prdx6 bands to the intensity obtained for α-tubulin. Data are shown as percent of *Cftr* ^+/+^ treated with saline (mean±S.E., n = 5 for saline and n = 4 for LPS ). * P≤0.05

Colonization by *P. aeruginosa* is characteristic of CF lung disease. *P. aeruginosa* LPS is a well known physiological inducer of ROS production by neutrophils in lung. To evaluate the levels of Prdx6 expression during oxidative challenge induced by *P. aeruginosa*, mice received intratracheal instillation of either *P. aeruginosa* LPS or saline and lungs were analysed 24 h later. Results were comparable to those obtained after PQ challenge (Fig. 6), i.e. Prdx6 expression was more strongly decreased after LPS (4-fold, *P* = 0.05) in *Cftr* ^−/−^ lung than in *Cftr* ^+/+^ (1.4-fold decrease, *P* = 0.54). Because neutrophil activation in response to LPS is exaggerated in *Cftr* ^−/−^ lung [30] and results in higher ROS production, LPS challenge was not used in this study to compare the antioxidant ability of *Cftr* ^−/−^ and *Cftr* ^+/+^ lung.

### Different forms and post-translational modifications of Prdx6 after PQ challenge

**Figure 7:**
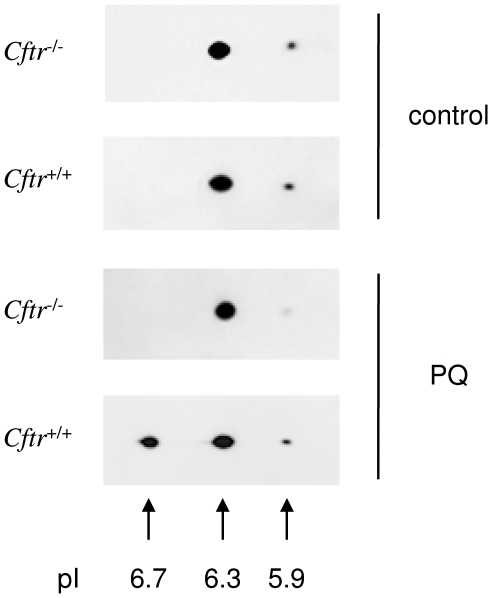
2D immunoblot analysis of Prdx6 forms after paraquat treatment. Lung protein extracts from *Cftr* ^+/+^ and *Cftr* ^−/−^ mice treated with either paraquat (PQ) or saline (control) were subjected to immunoblot analysis after IEF/SDS-PAGE. Two forms (pI ∼5.9 and ∼6.3) are present in *Cftr* ^+/+^ and *Cftr* ^−/−^ lung treated or not with PQ. An additional form of Prdx6 (pI ∼6.7) is only detected in lung extracts form *Cftr* ^+/+^ mice treated with PQ. For both conditions, immunoblots are representative of n = 4 animals of each genotype.

Equivalent levels of Prdx6 in *Cftr* ^+/+^ and *Cftr* ^−/−^ lung after exposure to PQ, together with a diminished capacity to eliminate PC-OOH in *Cftr* ^−/−^ mice suggest a compromised antioxidant function of Prdx6 that could be associated with post-translational modifications. In order to look for the presence of different forms of Prdx6 that could reflect such modifications, lung protein extracts from mice treated with either PQ or saline were subjected to 2D electrophoresis followed by immunoblotting with an anti-Prdx6 antibody (Fig. 7). 2D electrophoresis allowed to resolve three Prdx6 forms (pI ∼5.9, ∼6.3 and ∼6.7). pI 5.9 and 6.3 forms are detected in both mouse types and in both conditions. Interestingly, the pI 6.7 spot is present only in *Cftr* ^+/+^ lung after PQ treatment.

**Figure 8:**
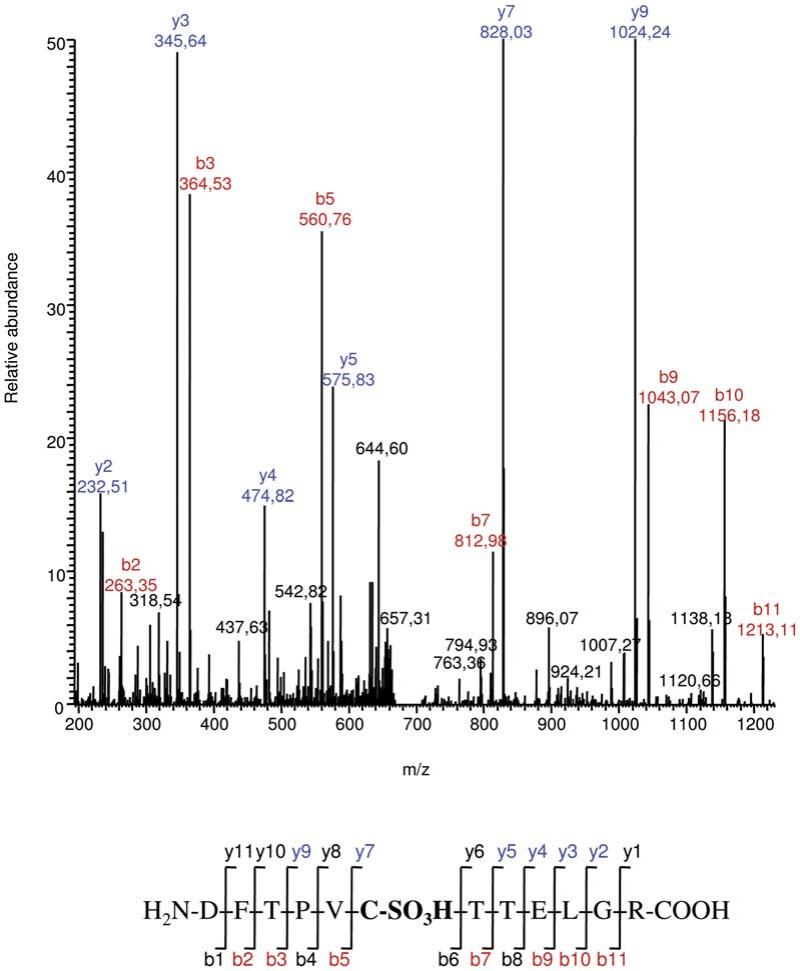
Cysteine 47 overoxidation to sulfonic acid in Prdx6 extracted from pI 6.3 spot. Fragmentation spectra of DFTPVC(SO3H)TTELGR peptide, corresponding to Prdx6 amino acid residues 42–53, unequivocally indicates oxydation of Cys47 to sulfonic acid. The most abundant peaks (singly charged) of the peptide are annotated as b- and y- series daughter ions, in red and blue respectively. Fragmentation spectra of the non-oxidized peptide show a similar fragmentation pattern, with a mass shift of 48 Da on peaks with oxidized cysteine (data not shown).

To characterize the different Prdx6 forms, spots corresponding to pI 6.3 and 6.7 were subjected to trypsin digestion and analyzed by mass spectrometry. The pI 5.9 spot was not analysed, as it was not detected by silver staining. Both spots were unambiguously identified as Prdx6 with high Mascot protein score and sequence coverage (Table 1). In the next step, error tolerant searches were performed for detection of post-translational modifications. An oxidative modification of Cys-47 (the cysteine of the active site for peroxidase function) to cysteine sulfonic acid (Cys47-SO_3_H) was identified only in the MS/MS spectrum from the pI 6.3 spot. The fragmentation spectrum (Fig. 8) of the observed peak (m/z = 639.81, doubly charged) matches the tryptic peptide DFTPVCTTELGR, corresponding to Prdx6 amino acid residues 42 to 53, plus three oxygen atoms. This spectrum provided enough sequence information for unequivocal assignment of Cys-47 oxidation to cysteine sulfonic acid. Despite a better Prdx6 sequence coverage for the pI 6.7 compared to the pI 6.3 spot, neither parent nor daughter ions corresponding to the peptide bearing the Cys47-SO_3_H modification were detected in the pI 6.7 spot. These data indicate that the PQ-induced population of Prdx6 observed only in *Cftr* ^+/+^ mice does not contain the overoxidized cysteine, but only its reduced form.

**Table 1:** Tandem mass spectrometry identification of Peroxiredoxin 6 in pI 6.3 and 6.7 spots.

pI of the spot	Database accession no	Protein name	Mascot protein score	Sequencing attemps	Sequence coverage
6.3	gi3219774	Peroxiredoxin 6	1012	129	76
6.7	gi3219774	Peroxiredoxin 6	2009	233	87

a Accession numbers were derived from the NCBInr database (version 20080624).

## Discussion

The diversity of cellular abnormalities associated with CFTR deficiency suggest that CFTR is more than a chloride channel and is somehow connected to a plethora of functions, as indicated by recent proteomic investigations of CF models [31]. Recent evidence showing that CFTR expression and function are modulated by oxidative stress [32] [33] [34] and that CFTR controls extracellular glutathione levels [8] [9] [10] suggest that loss of CFTR function may result in a poor adaptive response to oxidants. Until now, most studies dealing with oxidative stress in CF lung have focused on the oxidant and antioxidant status of the extracellular compartment. The current study presents evidence of higher levels of intracellular ROS and lipid peroxidation in basal conditions, despite a strong enzymatic antioxidant response mobilizing SOD, GPx, and, more particularly, Prdx6. *In vivo*, oxidative stress challenge induced by paraquat unmasks an increased susceptibility to phospholipid peroxidation and protein carbonylation in the lung from mice lacking CFTR. In addition, abnormal post-translational modifications of Prdx6 in response to PQ-induced oxidative stress in *Cftr* ^−/−^ cells might explain their compromised ability to reduce PL-OOH.

Our results clearly indicate that important alterations in both oxidant and antioxidant status of airways, and subsequent oxidative damage, are early events in the life of *Cftr* ^−/−^ mice and provide evidence of moderate oxidative stress in the lung of mice lacking CFTR. In airway cells freshly isolated from *Cftr* ^+/+^ and *Cftr* ^−/−^ mice, steady state levels of intracellular ROS, *i.e.* H_2_O_2_, are higher in the latter (Fig. 1A). Comparable results have been reported by Day *et al* [35] who used the H_2_DCF-DA probe to evaluate H_2_O_2_ levels in the human cell line IB3 and its CFTR-corrected counterpart, S9. Under this pro-oxidative environment, enhanced oxidative damage can be expected, since H_2_O_2_ is supposed to give rise to the highly reactive and deleterious hydroxyl radical (OH^•^) through the Fenton reaction [36]. Accordingly, we found lipid peroxidation, as measured by the TBARS assay, slightly but significantly higher in lung tissue from *Cftr* ^−/−^ mice as compared to their *Cftr* ^+/+^ littermates (Fig. 1B).

In contrast to the TBARS assay, direct HPLC analysis of phospholipid hydroperoxides did not give any indication of increased lipid peroxidation in *Cftr* ^−/−^ lung. The lack of correlation between the two methods is not surprising and may result from the fact that PL-OOH are labile and rapidly decomposed, unlike the more stable MDA, formed via the decomposition of lipid peroxide products, which is used as a reliable index of lipid peroxidation.

Lipid peroxidation in *Cftr* ^−/−^ lung is likely to be a consequence of increased intracellular ROS, even in the absence of any detectable extracellular sources, like neutrophil infiltration or bacterial colonization. It has been recently shown that dysfunctional CFTR leads to a mitochondrial oxidative imbalance that appears to be a consequence of lower mitochondrial GSH levels [35]. The unopposed mitochondrial-derived ROS could then contribute to oxidative damage to lipids and other cell macromolecules.

High levels of intracellular pro-oxidant compounds generally lead to an adaptive response that results in stimulation of antioxidant enzymes, like SOD, GPx or CAT, which provide cell protection against oxidative damage [37]. We have shown elevated levels of both SOD and GPx activities, with a much more pronounced -fold increase in SOD compared to GPx in lung from *Cftr* ^−/−^ mice (Fig. 2). The ratio between the activity of the first (SOD) and second (GPx) step of antioxidant defence, rather than the absolute levels of these enzymes *per se*, may represent an important determinant of antioxidant function [38]. Taking into account this physiologically critical role of the balance between both enzymes, the significant increase in SOD/GPx ratio observed in *Cftr* ^−/−^ lung, mainly due to a drastic increase in SOD activity, may favor O_2_^•−^ to H_2_O_2_ conversion leading to H_2_O_2_ accumulation. These enzymatic data are consistent with the observed increase in intracellular ROS in *Cftr* ^−/−^ cells (Fig. 1).

We have evaluated the *in vivo* capacity of lung to respond to oxidative insult induced by paraquat, which essentially targets this organ [29]. PQ administration resulted in a higher level of phospholipid peroxidation (Fig. 4) as well as protein carbonylation in *Cftr* ^−/−^ lung compared to *Cftr* ^+/+^. Since intracellular ROS levels were comparable in *Cftr* ^−/−^ *vs Cftr* ^+/+^ airway cells after PQ (data not shown), we assume that increased phospholipid peroxidation was due to a marked decreased ability of *Cftr* ^−/−^ cells to limit phospholipid peroxidation. This phenomenon probably results from an inefficient adaptive response to an oxidative burden. The failure to fully protect from oxidative injury is likely to occur during CF infection/inflammation, when activated neutrophils produce high levels of ROS. Lipid peroxidation products, such as MDA, can strongly activate redox-dependant pathways that increase inflammatory cytokine production and contribute to the exaggerated inflammatory responses in CF.

Among lung peroxidases, Prdx6 has the unique ability to reduce PL-OOH, as other PL-OOH reductase enzymes have limited or no activity in this tissue [39] [40] [41]. It was therefore expected that this antioxidant would be basally activated in *Cftr* ^−/−^ cells. Accordingly, quantitative RT-PCR and immunoblot analyses showed a significant and similar increase of Prdx6 expression at the mRNA and protein levels in *Cftr* ^−/−^ lung (Fig. 3), suggesting that upregulation of Prdx6 is mainly controlled at the mRNA level. In alveolar type II cells isolated from adult rats subjected to hyperoxia, mRNA and protein levels of Prdx6 increased by 1.5- to 2-fold [42]. An opposite down-regulation of Prdx6 expression has recently been observed in the liver of a mouse model of chronic ethanol consumption [43]. Interestingly, Pak *et al* [44] have shown that mRNA and protein levels of Prdx6 are increased in human lens epithelial cells in response to moderate concentrations of H_2_O_2_, but decreased below control levels when a higher dose of H_2_O_2_ is used.

In the present study, a similar dose-dependent reverse effect exerted by ROS might explain the higher Prdx6 expression level observed in *Cftr* ^−/−^ cells in basal conditions, as well as the down-regulation of the enzyme in both *Cftr* ^−/−^ and *Cftr* ^+/+^ after oxidative challenge (Fig. 5 and 6). By triggering a PMN response, LPS instillation is a strong inducer of ROS production in the lung. Our finding that both LPS and PQ challenges have a similar effect on Prdx6 expression suggests that the dramatic down-regulation of Prdx6 expression induced by PQ in *Cftr* ^−/−^ lung is not specifically related to PQ and may occur during lung infection by *P. aeruginosa*. In addition, the PQ challenge experiment suggests that Prdx6 expression in response to oxidant is in part regulated at the mRNA level (Fig. 5).

The regulation of the *Prdx6* gene has recently been investigated in mouse liver [45] and in the human lung epithelial cell line A-549 [46]. In the latter, *Prdx6* expression is up-regulated by the redox-sensitive transcription factor Nrf-2, which binds a functional antioxidant response element (ARE) on the *Prdx6* promoter. Since CF epithelial cells exhibit significantly lower expression and transcriptional activity of Nrf-2, as demonstrated by a recent study [47], it is unlikely that Nrf-2/ARE is responsible for the increased basal expression of *Prdx6* mRNA in *Cftr* ^−/−^ cells. Conversely, it has been shown that NF-κB inhibition leads to a marked increase in *Prdx6* mRNA [45]. Such an inverse relationship between NF-κB activation and *Prdx6* expression has also been reported by Roede *et al* [43] in a murine model of chronic ethanol consumption. Due to the fact that both *in vivo* PQ administration [48] and LPS challenge induce a strong and sustained increase in NF-κB activation in the lung, it is reasonable to suggest that NF-κB may represent a major player in the down-regulation of Prdx6 expression consecutive to PQ and LPS treatments. A number of publications have reported an abnormal NF-κB regulation in CF airway cells [49] [50] [51]. For instance, *Cftr*-knockout mice challenged by intratracheal instillation of *P. aeruginosa* fail to regenerate I-κB alpha, once it is degraded. As a consequence, they show an excessive and prolonged lung activation of NF-κB [50].

The greater fold decrease in Prdx6 expression after PQ in *Cftr* ^−/−^ lung leads to a Prdx6 expression level equivalent to that in *Cftr* ^+/+^, without any change in subcellular localization. However *Cftr* ^−/−^ lung presents a lower ability to limit phospholipid peroxidation. It can be therefore hypothesized that *Cftr* ^−/−^ lung presents a compromised Prdx6 peroxidase function resulting from protein modification. To test this hypothesis, we performed 2D electrophoresis and mass spectrometry analysis. 2D analysis did not allow to resolve differentially expressed Prdx6 populations in resting conditions, as two main Prdx6 spots ( pI 5.9 and pI 6.3) were detected in both *Cftr* ^+/+^ and *Cftr* ^−/−^ lung extracts (Fig. 7). By contrast, from the same analysis after lung exposure to PQ resolved a Prdx6 population, at pI 6.7, appearing solely in *Cftr* ^+/+^. Mass spectrometry analysis indicated that the PQ-induced Prdx6 population contains the reduced cysteine 47 but not its sulfonylated form that is present in the pI 6.3 spot (Fig. 8). Overoxidation of this redox-active cysteine to a sulfinic or sulfonic derivative is supposed to be irreversible *in vivo* [52], resulting in a non-functional enzyme [53] [54] [55]. If the PQ-induced Prdx6 population in *Cftr* ^+/+^ cells corresponds to an active form, this population would decrease the proportion of overoxidised inactive forms of Prdx6 in *Cftr* ^+/+^ lung. Biochemical characterisation of the stress-induced Prdx6 population at pI 6.7 is currently under investigation. We can speculate that this stress-induced Prdx6 population may represent an adaptive modification of the Prdx6 protein to limit its overoxidation and to maintain its antioxidant activity in response to a strong oxidative burden. An example of modification has been recently reported by Parmigiani *et al* [56] for other two members of the peroxiredoxin family. The authors show that acetylation of Prdx1 and Prdx2 increases their activity and their relative resistance to overoxidation. Further studies are needed to determine how a CFTR defect contributes to abnormal Prdx6 modification in response to oxidative stress.

In conclusion, our results indicate that despite a significant activation of the main cellular antioxidant defences, the lung of *Cftr* ^−/−^ mice presents higher levels of ROS and lipid peroxidation products. Importantly, attenuation of lung antioxidant defences by a significant decrease in Prdx6 expression and abnormal post-translational modification during an additional oxidative stimulus, strongly enhances phospholipid peroxidation. Prdx6 failure may represent an important underlying mechanism in the progression of epithelial dysfunction in CF in response to oxidative insult.
